# Supplementary material for: Indian Ocean warming as a driver of the North Atlantic warming hole
Source: Nat Commun. 2020 Sep 22;11:4785. doi: 10.1038/s41467-020-18522-5 (PMC7509804; doi:10.1038/s41467-020-18522-5)
Supplement: Supplementary file 1 — Supplementary Information [file 41467_2020_18522_MOESM1_ESM.pdf]

Supplementary Information for:

Indian Ocean warming as a driver of the North Atlantic warming hole

Shineng Hu[1,2*] and Alexey V. Fedorov[3,4]

[1] *Lamont-Doherty Earth Observatory of Columbia University, Palisades, NY, USA*

[2] *Division of Earth and Ocean Sciences, Nicholas School of the Environment, Duke University, Durham, NC, USA*

[3] *Department of Earth and Planetary Sciences, Yale University, New Haven, CT, USA*

[4] *LOCEAN/IPSL, Sorbonne University, Paris, France*

The Supplementary Materials include:

Supplementary Fig. 1: Observed trends in surface heat fluxes over the North Atlantic.

Supplementary Fig. 2: Quasi-stationary atmospheric Rossby wave trains generated by tropical Indian Ocean (TIO) warming.

Supplementary Fig. 3: Fast response of surface turbulent heat flux to tropical Indian Ocean (TIO) warming.

Supplementary Fig. 4: Observed North Atlantic climate anomalies associated with relative tropical Indian Ocean (TIO) warming.

*Corresponding author: Shineng Hu (shineng@ldeo.columbia.edu)

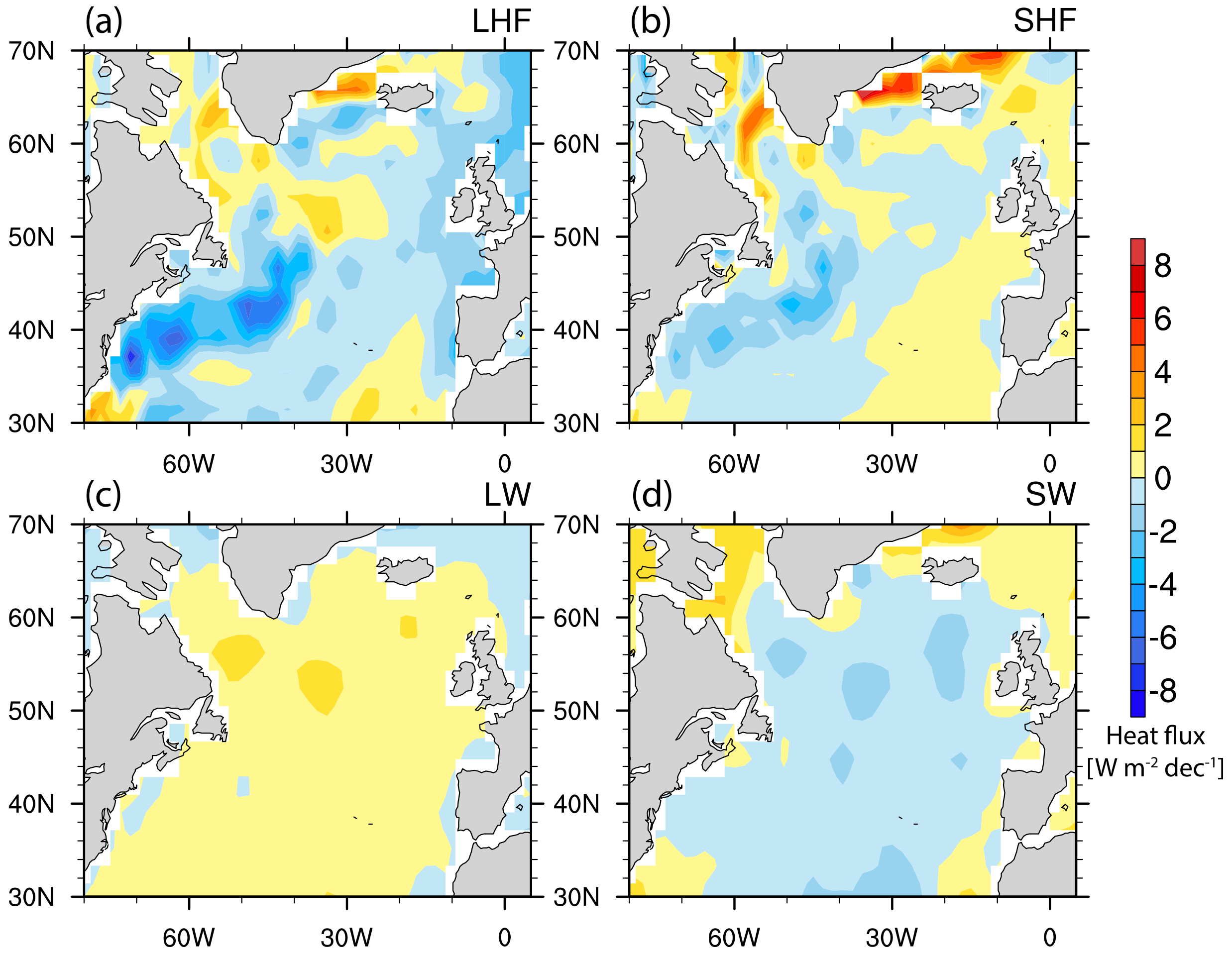

Supplementary Fig. 1: Observed trends in surface heat fluxes over the North Atlantic. Long-term trends during 1950-2015 in surface (a) latent heat flux (LHF; units: W m^{-2} decade^{-1}), (b) sensible heat flux (SHF; units: W m^{-2} decade^{-1}), (c) longwave radiative flux (LW; units: W m^{-2} decade^{-1}), and (d) shortwave radiative flux (SW; units: W m^{-2} decade^{-1}). All the surface heat flux terms are defined as downward positive. NCEP/NCAR Reanalysis 1 is used.

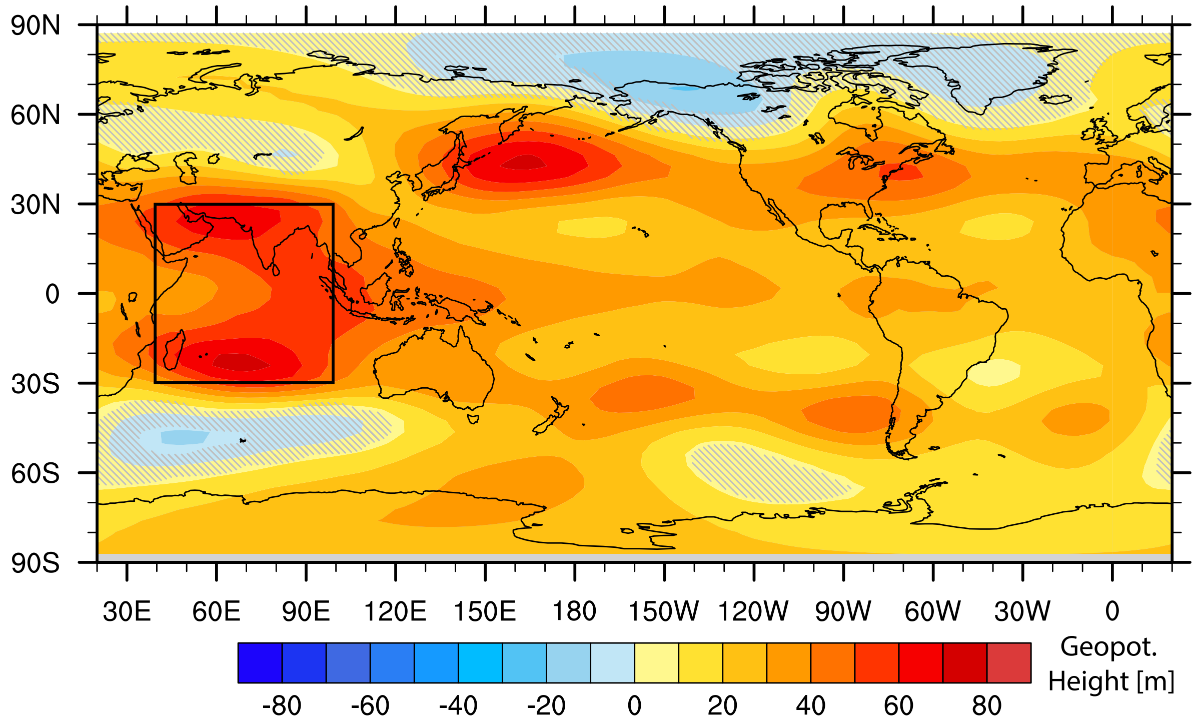

Supplementary Fig. 2: Quasi-stationary atmospheric Rossby wave trains generated by tropical Indian Ocean (TIO) warming. Anomalies in geopotential height (units: m) at the level of 193 of CESM's hybrid vertical coordinate, close to 200 mb. Anomalies are averaged within the first 40 years of the integration (i.e. the initial response) for the TIO+1C experiment with respective to the PI simulation. Grey hatches highlight the areas of lower statistical significance (p-values of Student's t-test above 0.05). The black box indicates the region where sea surface temperature anomalies are imposed in the TIO+1C and other idealized experiments.

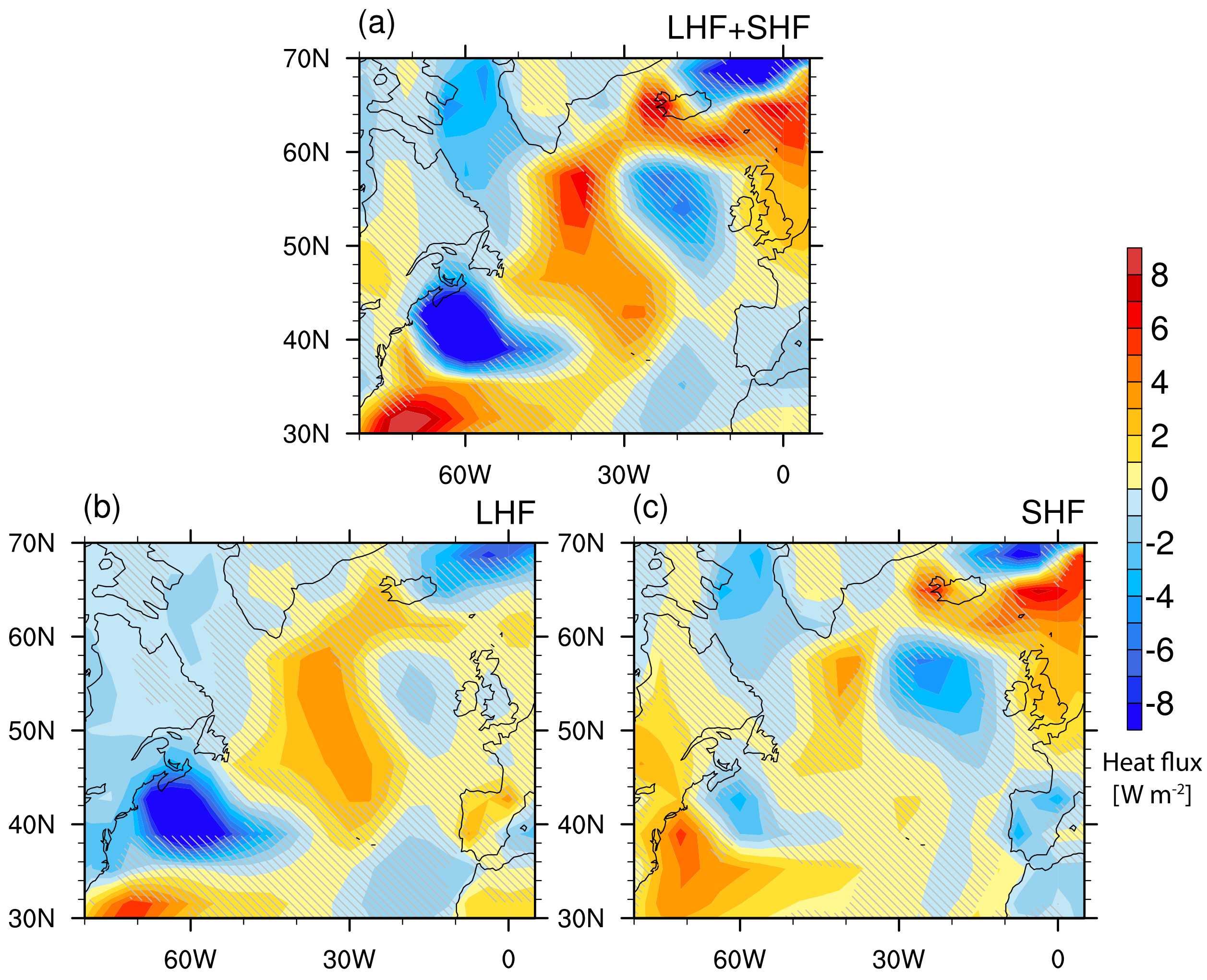

Supplementary Fig. 3: Fast response of surface turbulent heat flux to tropical Indian Ocean (TIO) warming. Anomalies in (a) total surface turbulent heat flux (units: W m^{-2}), (b) surface turbulent latent heat flux (LHF; units: W m^{-2}), and (c) surface turbulent sensible heat flux (SHF; units: W m^{-2}), for the TIO+1C experiment with respective to the PI simulation, averaged for Years 1-40. Grey hatches highlight the areas of lower statistical significance (p-values of Student's t-test above 0.05).

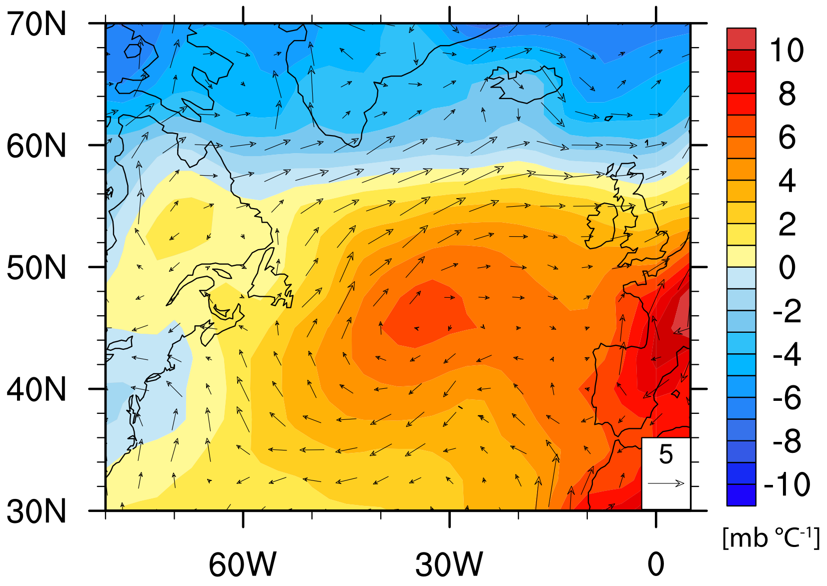

Supplementary Fig. 4: Observed North Atlantic climate anomalies associated with relative tropical Indian Ocean (TIO) warming. The plot shows a linear regression of sea level pressure (SLP; units: mb $°C^{-1}$) and surface wind vectors (units: m s^{-1} $°C^{-1}$) onto the relative TIO sea surface temperature (SST). A 11-year running mean is applied to all variables before the regression analysis is conducted. The relative TIO SST is defined as average SST in the Indian Ocean (30°S-30°N, 40°E-100°E) minus average SST in the whole tropical ocean (30°S-30°N). The surface wind and SLP data are from NCEP/NCAR Reanalysis 1, and the relative TIO SST is calculated based on ERSST v4.
